# Intercultural competence of Chinese students abroad: An investigation under Sino-foreign Cooperative Education Programs

**DOI:** 10.1371/journal.pone.0316937

**Published:** 2025-02-05

**Authors:** Pei Yang, Xiangge Zhao, Xinxin Zhang, Anran Li

**Affiliations:** School of Foreign Languages for International Business, Hebei Finance University, Baoding, Hebei, China; McGill University, CANADA

## Abstract

The rapid growth of Sino-Foreign Cooperative Education Programs highlights the critical role of intercultural competence (IC) for Chinese students studying abroad. While extensive research exists on domestic contexts, empirical studies on Chinese students’ experiences at foreign partner institutions remain scarce. This study addresses this gap by examining the intercultural competence of Chinese students in these programs and identifying key factors influencing their development. Using a mixed-methods approach, the study surveyed 130 Chinese students in Ireland and conducted WeChat video interviews with 16 participants. The results indicate that Chinese students generally exhibit moderately high levels of IC. Language proficiency, intercultural training, and intercultural contact experiences were significantly and positively associated with IC, while gender showed no significant correlation. These findings highlight the complex interplay of factors shaping intercultural competence in international education. The study offers valuable insights for educators and policymakers to improve intercultural training and support in Sino-Foreign Cooperative Education Programs. It also fills a key research gap by providing empirical evidence on Chinese students’ intercultural experiences abroad, laying the groundwork for future research and practical improvements.

## 1 Introduction

Since the Ministry of Education implemented the Regulations on Sino-Foreign Cooperative Education in 2003 [1], China’s higher education has entered a new phase of internationalization. These programs, combining resources and expertise from Chinese and foreign institutions, have become a crucial part of the higher education landscape, with 2,332 such programs and institutions established by the end of 2020 [2,3]. Many adopt “2+2” or “3+1” models, where students study two to three years in China before completing one to two years abroad to earn dual or joint degrees [4]. These programs aim to broaden students’ academic horizons and immerse them in intercultural learning. However, many students face difficulties in navigating cultural differences and communication barriers due to insufficient intercultural competence, leading to confusion and discomfort [5].

Intercultural competence, the ability to communicate effectively and appropriately with people from different cultural backgrounds [6], is vital for students in these programs. It significantly influences their academic success, adaptation to life abroad, and ability to build meaningful intercultural relationships [5]. Moreover, it is closely linked to the sustainability and quality of Sino-Foreign Cooperative Education Programs [7]. Despite meeting language proficiency requirements, many students struggle with cultural adaptation, revealing gaps in their intercultural competence [8].

Previous research has primarily focused on students’ experiences within domestic settings during their studies in China, examining aspects such as curriculum design, administrative challenges, and academic outcomes [9–11]. However, there is limited empirical research on Chinese students’ intercultural competence while studying abroad [12,13]. In foreign environments, students face heightened intercultural challenges, making intercultural competence crucial for their adaptation [14]. Additionally, existing studies are often theoretical or descriptive [15], lacking empirical data on students’ actual intercultural experiences and competence abroad. Addressing this gap is vital for understanding how students adapt to foreign academic environments and improving the effectiveness of international programs.

This study explores the intercultural competence of Chinese students in Sino-Foreign Cooperative Education Programs, focusing on their experiences at foreign partner institutions. Using a mixed-methods approach, it examines how students navigate intercultural challenges and identifies key factors influencing their competence development. The findings aim to inform strategies for improving support systems and educational practices, enhancing both the sustainability and effectiveness of these programs while contributing valuable insights to research and practice.

## 2 Literature review

### Concepts and assessment of intercultural competence

Intercultural competence emerged in the 1970s as scholars recognized the need for effective communication across cultures in a globalized world [16]. However, defining it remains challenging due to its complexity [17]. [18] identified four key aspects: knowledge, skills, attitudes, and awareness. [19] defined it as “the ability to communicate effectively and appropriately in intercultural situations based on one’s intercultural knowledge, skills, and attitudes” [p.33], emphasizing its practical application. The framework of [20], which includes attitudes, knowledge, interpreting skills, discovery skills, and critical cultural awareness, is influential in language teaching and intercultural communication fields [21–23].

Assessing intercultural competence is complex due to methodological challenges [24]. Existing models often overlap, making distinctions difficult. Tools like the Behavioral Assessment Scale for Intercultural Competence (BASIC) [25], the Assessment of Intercultural Competence (AIC) [26], and the Intercultural Development Inventory (IDI) [27] are based on Western perspectives, with fewer models reflecting non-Western views. For Chinese students, the Assessment of Intercultural Competence of Chinese College Students (AIC-CCS) proposed by [28] is a significant contribution, addressing gaps in Chinese IC research. Qualitative methods, such as interviews and portfolio assessments, provide a deeper understanding of intercultural competence [29]. Recent studies advocate for mixed-methods approaches combining quantitative and qualitative data for a comprehensive assessment [30–32].

### Factors influencing intercultural competence

Research has shown that various demographic, behavioral, and institutional factors influence students’ intercultural competence. For instance, gender is often correlated with intercultural competence, with women generally displaying higher intercultural sensitivity [19,33]. Language proficiency also plays a crucial role, as [34] found that language skills and intercultural interactions mutually reinforce each other, enhancing intercultural competence. [35] further observed that advanced language skills help individuals better understand cultural nuances, enabling more effective interactions. High language proficiency is also linked to greater empathy and openness to diverse perspectives, key traits for developing intercultural competence [36].

At the behavioral level, intercultural contact plays a key role in developing intercultural competence. Such contact, whether direct or indirect, involves interactions between individuals from different cultural backgrounds [37]. Studies show that it fosters cultural learning and perspective-taking, enhances intercultural competence, boosts language learning motivation, and reduces anxiety [38]. This aligns with [39]’s findings on its positive impact on openness to cultural diversity and language learning. In China, [40] developed a 31-item intercultural contact scale across six factors: Domestic Social Media (DSM), Foreign Social Media (FSM), Domestic Intercultural Communication Activity (DICA), Foreign Intercultural Communication Activity (FICA), Cultural Products (CP), and Multimedia and Courses (MMC). Their research confirmed that both direct and indirect contact positively influence intercultural competence, consistent with Kormos and Csizér’s framework [37].

Intercultural training is widely recognized as essential for developing students’ intercultural competence at the institutional level. Receiving training both before and after study abroad programs significantly enhances intercultural competence [41]. [42] recommended experiential training methods, such as role-playing and case studies, to improve intercultural competence among American and German MBA students. [43] emphasized the importance of intercultural training and suggested ten best practices, including using longer, more comprehensive training and providing developmental feedback throughout the process.

### Intercultural competence in Sino-Foreign Cooperative Education Programs

Sino-Foreign Cooperative Education Programs, launched by the Chinese government, are collaborations between Chinese and foreign higher education institutions [44]. These programs enable students to earn degrees or credits from both institutions without establishing new entities [45]. Initiated in 2003 under the Ministry of Education’s regulations, they have expanded rapidly, with over 2,300 programs by 2020, reflecting the internationalization of Chinese higher education [3].

Unlike regular international students, participants in these programs benefit from resources and support from both their home and host universities [46]. Typically, they study abroad for at least a year, facing unique cultural adaptation challenges and balancing academic requirements from both institutions [47]. Intercultural competence is critical in these programs, as it helps students navigate cultural differences, enhance academic and social experiences, and contribute to the programs’ success [48–50].

However, most research has focused on Chinese students’ intercultural competence during their domestic studies [9–11]. While studies like [2,4] have explored curriculum design, administrative challenges, and academic performance, they lack empirical data on students’ intercultural experiences. Few studies, such as [51]’s survey on intercultural sensitivity and [52]’s analysis of self-reported intercultural challenges, address students’ intercultural competence in China, leaving the study abroad phase largely unexplored. This phase is crucial, as students face more intense challenges [53]. Understanding how they adapt and develop intercultural competence is essential [54].

Existing research often overlooks factors such as prior intercultural exposure and language proficiency, which are vital for supporting students’ adaptation abroad [34–36,55]. Addressing these gaps will enhance understanding and improve strategies for fostering intercultural growth in Sino-Foreign Cooperative Education Programs.

The discussions above lead to the central research questions this study seeks to address:

RQ1: What is the current state of intercultural competence among Chinese students participating in Sino-Foreign Cooperative Education Programs, particularly during their time at foreign partner institutions?

RQ2: What are the key factors influencing the development of intercultural competence among these students, especially how do gender, intercultural training, language proficiency and intercultural contact experience contribute to their intercultural competence?

The findings of the study are expected to offer insights into improving educational strategies and support in Sino-Foreign Cooperative Education Programs, enhancing their effectiveness and sustainability. Additionally, the study provides empirical evidence on the intercultural experiences and competence of Chinese students in these programs, addressing a critical gap in the research.

## 3 Methods

### Research design

**Fig 1:**
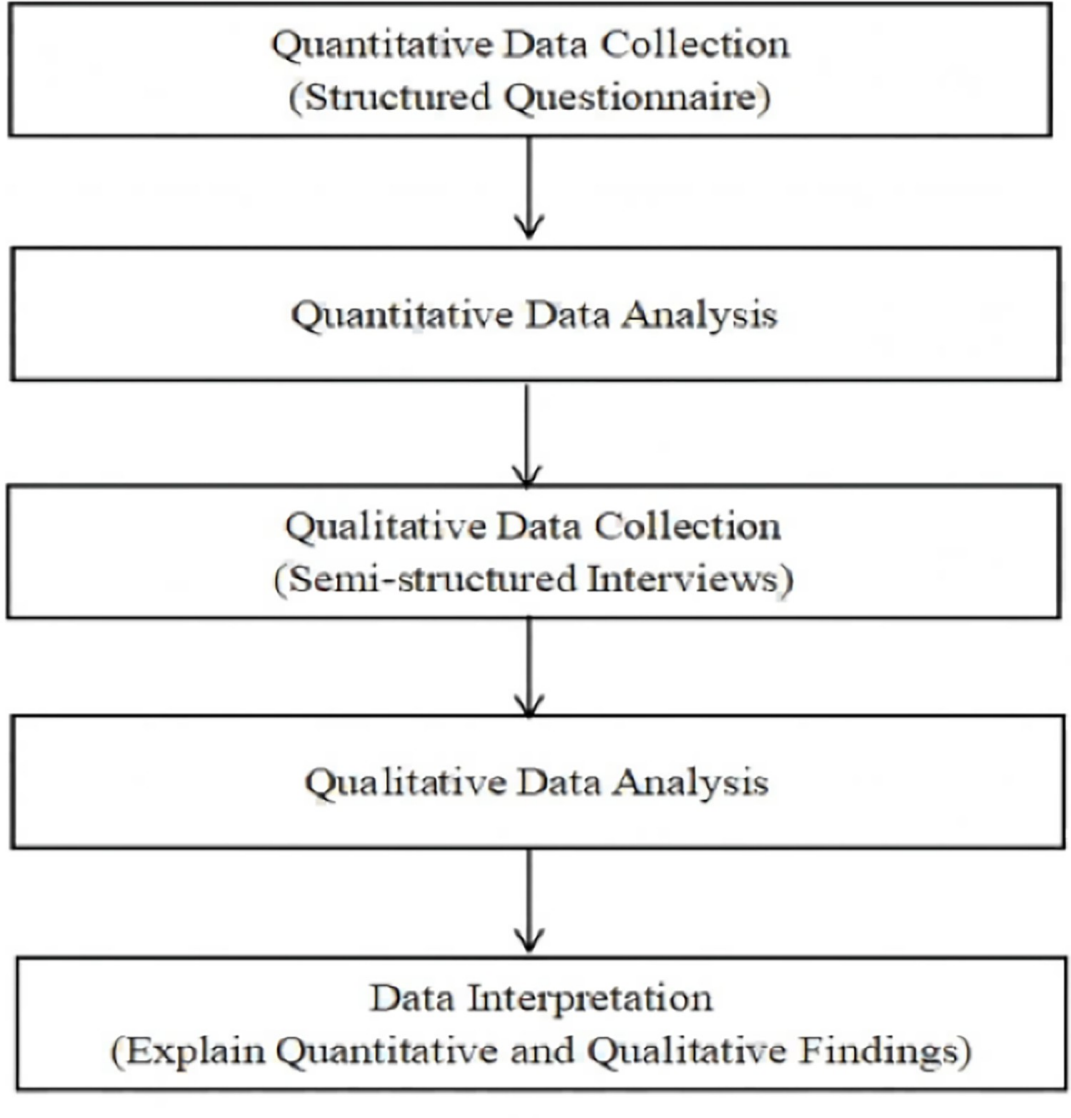
The sequential mixed methods design.

This study utilized a sequential explanatory mixed research method design to gain comprehensive insights into the development and influencing factors of intercultural competence among Chinese students in Sino-Foreign Cooperative Education Programs. It involves collecting and analyzing quantitative data first, followed by the collection and analysis of qualitative data to help explain confusing, contradictory or unusual survey responses [56]. Fig 1 illustrates the process of the sequential mixed methods design.

In the first phase, quantitative data were collected using structured questionnaires distributed to Chinese students studying abroad under Sino-Foreign Cooperative Education Programs. Statistical analyses, including independent t-tests, one-way ANOVA, and Spearman’s Rank-Order Correlation, were conducted to assess the students’ levels of intercultural competence and identify influencing factors. Based on the quantitative results, respondents were selected for semi-structured interviews in the second phase. These interviews offered deeper insights into the students’ experiences and perceptions regarding their intercultural competence, helping to contextualize and explain the quantitative findings. Finally, the integration of quantitative and qualitative results was conducted in the Discussion section to provide a comprehensive understanding of the findings.

### Research instruments

A questionnaire survey was conducted with Chinese students studying in Ireland to gather data. The first section collected demographic information, such as gender, age, grade, English test scores, and intercultural training experience. The second section adapted a scale by [40] to assess students’ intercultural contact, focusing on Foreign Social Media (FSM) and Foreign Intercultural Communication Activity (FICA) with nine items (FSM1-3, FICA1-6). The third section used the Assessment of Intercultural Competence of Chinese College Students (AIC-CCS) by [28]. Sections II and III used a 5-point Likert scale, from 1 (strongly disagree) to 5 (strongly agree). These two scales were chosen for their specific design tailored to Chinese students, their high validity and reliability [28,57], and their frequent use in studies on Chinese students’ intercultural competence [58–60].

Semi-structured interviews were conducted to further explore the development of Chinese students’ intercultural experiences. Sixteen participants were selected based on the results from Section II of the questionnaire survey, evenly distributed across four proficiency levels: extremely high, high, medium, and low. The interviews, followed a predefined protocol to ensure consistency and relevance to the research objectives. Before the interviews, the concept and components of intercultural competence were explained to participants. The protocol included two primary questions: (1) “How do you perceive your intercultural competence while abroad?” and (2) “What factors do you believe influence your intercultural competence?” These questions were carefully designed to align with the research objectives and elicit insights into participants’ experiences and perceptions.

### Participants

The study focused on Chinese students in Sino-Irish Cooperative Education Programs in Ireland. Participants were recruited through convenience and snowball sampling. Initially, 50 students from the principal researcher’s institution received the questionnaire on November 15, 2023. These students were asked to share the link with classmates and friends in similar programs. By December 31, 2023, 145 students had completed the questionnaire, yielding 130 valid responses. Table 1 summarizes the demographic details of these respondents, including gender, age, grade, English test scores, and intercultural training experience.

**Table 1:** Participants’ demographics.

Demographics		Number	Percentage
Gender	male	57	43.85%
	female	73	56.15%
Age	20	15	11.54%
	21	38	29.23%
	22	45	34.62%
	23	23	17.69%
	24	9	6.92%
Grade	two	23	17.69%
	three	65	50.00%
	four	42	32.31%
IELTS scores	5	6	4.62%
	5.5	28	21.54%
	6	34	26.15%
	6.5	37	28.46%
	7	19	14.62%
	7.5	6	4.62%
Intercultural training experience	With intercultural training experience	64	49.23%
	Without intercultural training experience	66	50.77%

The sample included 57 male (43.85%) and 73 female (56.15%) students. Most were aged 21 (29.23%, 38 students) or 22 (34.62%, 45 students), followed by 23 (17.69%, 23 students), 20 (11.54%, 15 students), and 24 (6.92%, 9 students). By grade, 23 students (17.69%) were in their second year, 65 (50%) in their third year, and 42 (32.31%) in their fourth year. English proficiency scores, converted to IELTS equivalents, showed most students scored 6.5 (28.46%, 37 students) or 6.0 (26.15%, 34 students), with smaller groups scoring 5.0 (4.62%, 6 students), 5.5 (21.54%, 28 students), 7.0 (14.62%, 19 students), and 7.5 (4.62%, 6 students). Additionally, 64 students (49.23%) had prior intercultural training, while 66 (50.77%) did not.

### Data collection and analysis

#### Quantitative data collection and analysis

The questionnaire was created using Wenjuanxing, a Chinese online survey platform, and distributed via a WeChat link. After receiving ethics approval from the School of Foreign Languages for International Business, Hebei Finance University (June 28, 2023), participants were informed about the study’s purpose, procedures, and their rights through a video shared on WeChat. They were assured of confidentiality and data security and provided electronic consent to participate, allowing interview recordings and the use of their data for publication. Participants then completed the questionnaire through the WeChat link, with pseudonyms used to ensure anonymity.

Quantitative data from the questionnaires were analyzed using SPSS 29.0. To address research question one-assessing the intercultural competence of Chinese students in Sino-Foreign Cooperative Education Programs-descriptive analysis was conducted, calculating frequencies, means, and standard deviations (SD). Intercultural competence was evaluated using the AIC-CCS questionnaire and the fuzzy comprehensive evaluation (FCE) model developed by [61]. The FCE model, suited for this study, provides a detailed assessment by examining weighted distributions across descriptive items and six core dimensions, offering a comprehensive view of participants’ intercultural competence.

To address the second research question-identifying key factors influencing intercultural competence, a multifaceted analysis was conducted. Independent t-tests examined the impact of gender and intercultural training, while one-way ANOVA analyzed the effect of language proficiency. Spearman’s Rank-Order Correlation was used to assess the relationship between intercultural contact experiences and intercultural competence.

#### Qualitative data collection and analysis

Interviews were conducted one week after the questionnaire survey, guided by the quantitative results. Lasting about 15 minutes each, the interviews focused on perceptions of intercultural competence and its influencing factors. To encourage open expression, the interviews were conducted in Chinese [62], transcribed, and then translated into English. Thematic analysis was carried out independently by two coders. Based on the process of thematic analysis suggested by [63], the two coders first familiarized themselves with the data by repeatedly reading the transcripts, allowing inductive codes to emerge. These codes were systematically applied, organized into themes and subthemes, and refined for coherence and relevance. Discrepancies were resolved through discussion to ensure the reliability of the thematic framework.

#### Validity and reliability of the study

To ensure validity, this study employed triangulation and peer debriefing [56]. Triangulation involved using data from multiple sources-questionnaires, interviews, and journals-with each research question supported by at least two instruments. Peer debriefing included a review by an experienced mixed-methods researcher, leading to revisions in the questionnaires, while interview and journal guidelines required no changes. Two translation specialists from Beijing Foreign Studies University reviewed the translations for clarity and accuracy.

Reliability of quantitative instruments was ensured through a pilot study with 20 participants who completed the questionnaires online. SPSS 26.0 analysis showed Cronbach’s Alpha indices of 0.856 for the intercultural contact scale and 0.912 for the AIC-CCS, indicating high reliability.

For qualitative data, inter-rater reliability was assessed. After training on research goals and the theoretical framework, the researcher and a second coder independently analyzed 20% of the journals and interview transcripts. The inter-rater reliability, measured by Cronbach’s α, was 0.868, exceeding the 0.70 threshold [64], confirming coding consistency.

## 4 Results

### Results for research question one

The AIC-CCS framework encompasses six key factors, each assessed through a total of 28 items (represented as ic1 to ic28). These factors are Knowledge of Self (KN1), Knowledge of Others (KN2), Intercultural Communicative Skills (SK1), Intercultural Cognitive Skills (SK2), Attitude (AT), and Awareness (AW). Based on the weight distribution of the six dimensions and 28 items included in the AIC-CCS scale delineated by [61], each participant’s intercultural competence was calculated.

The analysis benchmark utilized in this study is based on the work of [65], in which a score below 3.00 indicates a low level of intercultural competence, scores between 3.00 and 3.50 represent a medium level, scores between 3.50 and 4.00 correspond to a high level, and scores above 4.00 signify an extremely high level of intercultural competence.

Table 2 below depicts the results of participants’ overall intercultural competence and its six dimensions with Mean, Standard Deviation and Variance. The current state of Chinese participants’ intercultural competence shows generally high levels across most dimensions. The mean score for overall intercultural competence (IC) suggests a high level of intercultural competence (M = 3.61) among the participants. Knowledge of self (KN1), intercultural communicative skills (SK1) and intercultural cognitive skills (SK2) also reflect high competence (M = 3.72, 3.59 and 3.57), underscoring the participants’ strong intercultural skills and knowledge of Chinese culture, despite the intermediately high scores for SK1 and SK2. The Attitude (AT) dimension stands out with an extremely high mean score (M = 4.08), indicating a particularly strong intercultural disposition among the students. However, knowledge of others (KN2) exhibits a moderate level of competence (M = 3.24), pointing to an area where there is room for improvement.

**Table 2:** Results of participants’ overall IC and its six dimensions.

Dimensions	N	Min	Max	Mean	Std. Deviation	Variance
Overall IC	130	2.00	5.00	3.61	0.52	0.27
KN1	130	2.33	5.00	3.72	0.65	0.42
KN2	130	1.30	5.00	3.24	0.63	0.40
AT	130	2.00	5.00	4.08	0.76	0.58
SK1	130	2.00	5.00	3.59	0.58	0.33
SK2	130	2.00	5.00	3.57	0.67	0.44
AW	130	2.00	5.00	3.79	0.69	0.47

In the qualitative interviews, most participants (12 out of 16) described their intercultural competence as moderate, which differs from the quantitative results. The remaining four participants, including two who rated their IC as medium and two as high in the questionnaire survey, assessed their IC as high in the interviews. Table 3 below exhibits the qualitative results of RQ1. The interviewees also discussed the reasons for their assessments, citing two main factors: first, insufficient knowledge of Irish culture; second, limited intercultural communication skills due to a lack of cultural exposure, leading to weak intercultural communication abilities.

**Table 3:** Qualitative results for RQ1.

Themes	Subthemes	Description	Example
Level of Intercultural Competence	Moderate	Interviewees describe their intercultural competence at a medium level	“I think my intercultural competence is just Okay. There’s still much room for improvement”.
	High	Interviewees comment their intercultural competence at a satisfactory level	“I think my intercultural competence is quite good”.
Reasons	Insufficient Irish Cultural Knowledge	Interviewees lack cultural knowledge	“I don’t think I gained enough knowledge of Ireland”.
	Limited Intercultural Communication Skills	Interviewees lack cultural exposure, resulting in poor intercultural communication skills	“I spent most of time with my friends from China. I don’t know how to communicate with foreigners”.

The results aligns with the moderate levels in knowledge of others (KN2) and intercultural skills (SK1, SK2) observed in the quantitative data.

### Results for research question two

#### Gender and intercultural competence

Table 4 below illustrates the results of the independent samples t-test for gender difference. The findings indicated that out of 130 participants, there were 73 females and 57 males. The analysis revealed no statistically significant difference in intercultural competence between male and female participants (t = -0.136, p > 0.05).

**Table 4:** Results of independent samples t-test by gender.

DV	IV	N	Mean	SD	*t*
Intercultural Competence	Male	57	3.6074	0.56	-0.136
	Female	73	3.6200	0.49	

#### Intercultural training and intercultural competence

The results of correlation between intercultural training and intercultural competence are presented in the following Table 5, indicating that there was a significant difference in intercultural competence between participants with intercultural training and without intercultural training (*t* = 2.442, *p*<0.05). Participants with intercultural training experience had higher scores of intercultural competencies than those without intercultural training experience.

**Table 5:** Results of correlation between intercultural training and IC.

DV	IV	N	Mean	SD	*t*
Intercultural Competence	With intercultural training	64	3.7261	0.55971	2.442*
	without intercultural training	66	3.5062	0.46359	

Note

*p< 0.05.

#### Language proficiency and intercultural competence

To examine the impact of language proficiency on Chinese participants’ intercultural competence, a one-way ANOVA test was conducted. Table 6 below presents the descriptive analysis of intercultural competence across different language proficiency levels. Participants with an IELTS score of 7.5 achieved the highest intercultural competence, with a mean of 4.45 (SD = 0.40), while those with an IELTS score of 5 had a lower mean of 3.72 (SD = 0.52). Participants with an IELTS score of 7 recorded a mean of 3.98 (SD = 0.44). Intermediate IELTS scores displayed varying results: participants scoring 5.5 had a mean of 3.34 (SD = 0.51), those scoring 6 had a mean of 3.51 (SD = 0.48), and those scoring 6.5 had a mean of 3.58 (SD = 0.40).

**Table 6:** Descriptive analysis results of IC by language proficiency.

DV	IV	N	Mean	SD
Intercultural competence	IELTS 5	6	3.72	0.52
	IELTS 5.5	28	3.34	0.51
	IELTS 6	34	3.51	0.48
	IELTS 6.5	37	3.58	0.40
	IELTS 7	19	3.98	0.44
	IELTS 7.5	6	4.45	0.40

The one-way ANOVA results in Table 7 below show a statistically significant difference in intercultural competence among participants with different levels of language proficiency, with an F-value of 8.847 and a p-value of less than .001.

**Table 7:** Comparative analysis results of IC by language proficiency.

	Sum of Squares	df	Mean Square	F	Sig.
Between Groups	9.276	5	1.855	8.847	< .001**
Within Groups	26.001	124	0.210		
Total	35.276	129			

Note

** p< 0.01.

**Fig 2:**
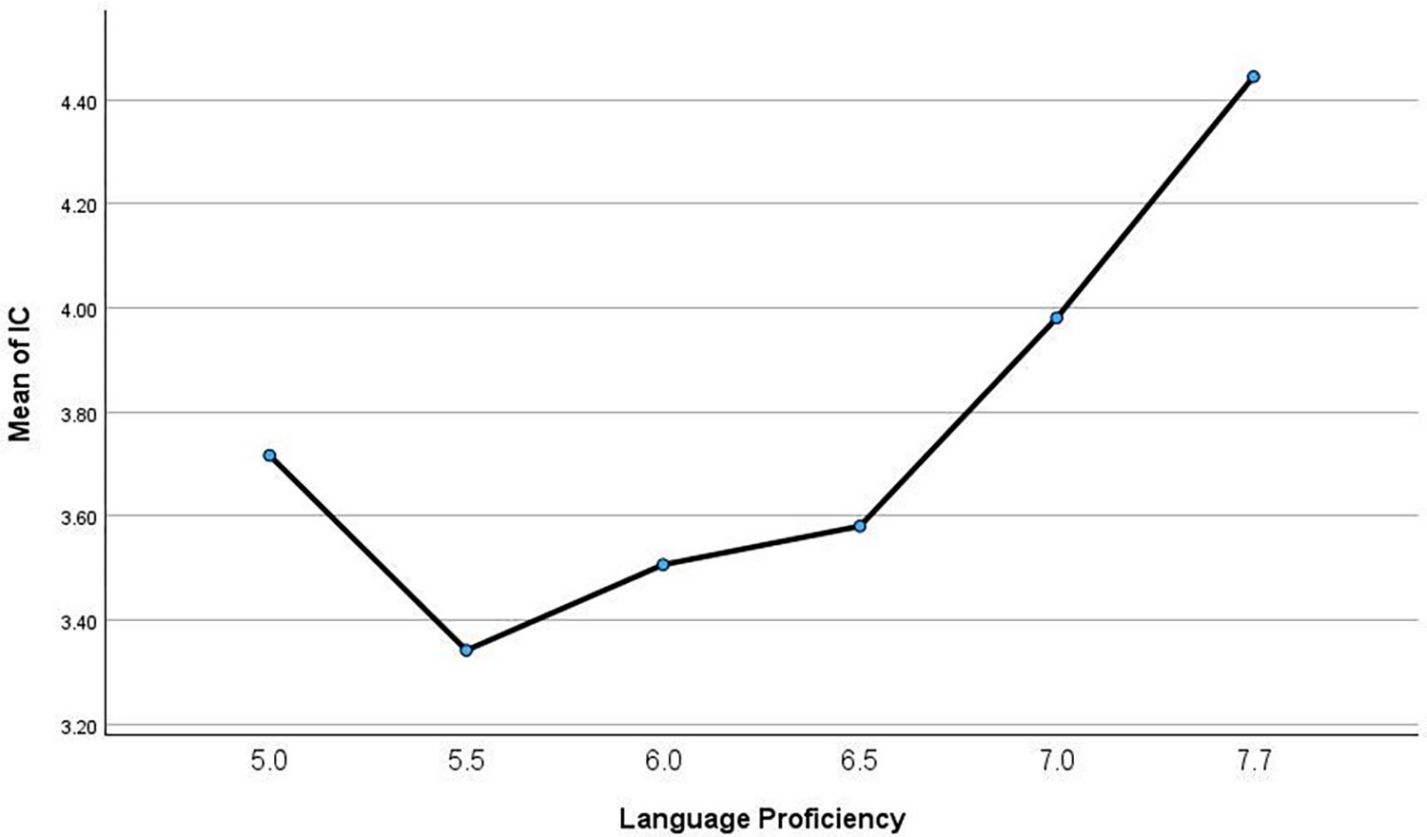
Post-hoc analysis of IC by language proficiency.

The post-hoc analysis in Table 8 revealed that participants with an IELTS score of 7.5 had significantly higher IC than those with lower scores, specifically those with scores of 5, 5.5, 6, and 6.5. This is visually supported by the steep upward trajectory in the graph for participants with higher IELTS scores as is shown in Fig 2.

**Table 8:** Post-hoc analysis of IC by language proficiency.

Multiple Comparisons							
Dependent Variable: IC							
	(I) Language Proficiency	(J) Language Proficiency	Mean Difference (I-J)	Std. Error	Sig.	95% Confidence Interval	
A						Lower Bound	Upper Bound
LSD	5.0	5.5	.37488	.20600	.071	-.0328	.7826
		6.0	.21020	.20277	.302	-.1911	.6115
		6.5	.13613	.20153	.501	-.2628	.5350
		7.0	-.26439	.21444	.220	-.6888	.1600
		7.5	-.72833*	.26438	.007	-1.2516	-.2051
	5.5	5.0	-.37488	.20600	.071	-.7826	.0328
		6.0	-.16468	.11686	.161	-.3960	.0666
		6.5	-.23875*	.11470	.039	-.4658	-.0117
		7.0	-.63927*	.13611	< .001	-.9087	-.3699
		7.5	-1.10321*	.20600	< .001	-1.5109	-.6955
	6.0	5.0	-.21020	.20277	.302	-.6115	.1911
		5.5	.16468	.11686	.161	-.0666	.3960
		6.5	-.07407	.10879	.497	-.2894	.1412
		7.0	-.47458*	.13116	< .001	-.7342	-.2150
		7.5	-.93853*	.20277	< .001	-1.3399	-.5372
	6.5	5.0	-.13613	.20153	.501	-.5350	.2628
		5.5	.23875*	.11470	.039	.0117	.4658
		6.0	.07407	.10879	.497	-.1412	.2894
		7.0	-.40051*	.12924	.002	-.6563	-.1447
		7.5	-.86446*	.20153	< .001	-1.2633	-.4656
	7.0	5.0	.26439	.21444	.220	-.1600	.6888
		5.5	.63927*	.13611	< .001	.3699	.9087
		6.0	.47458*	.13116	< .001	.2150	.7342
		6.5	.40051*	.12924	.002	.1447	.6563
		7.5	-.46395*	.21444	.032	-.8884	-.0395
	7.5	5.0	.72833*	.26438	.007	.2051	1.2516
		5.5	1.10321*	.20600	< .001	.6955	1.5109
		6.0	.93853*	.20277	< .001	.5372	1.3399
		6.5	.86446*	.20153	< .001	.4656	1.2633
		7.0	.46395*	.21444	.032	.0395	.8884

Therefore, both the statistical analysis and the visual data representation underscore a strong positive correlation between higher language proficiency and enhanced intercultural competence.

#### Intercultural contact and intercultural competence

A Spearman’s Rank-Order Correlation analysis was conducted to examine how Chinese participants’ intercultural contact experiences impact their intercultural competence. Intercultural contact was measured across two dimensions: Foreign Social Media (FSM) and Foreign Intercultural Communication Activity (FICA). The results of Spearman’s Rank-Order Correlation analysis are presented in the following Table 9, revealing a significant positive relationship between participants’ intercultural contact experiences and their intercultural competence (IC). The FSM dimension correlates positively with IC (r = 0.387, p < 0.01), indicating that greater engagement with foreign social media enhances IC. Sub-dimensions FSM1 (r = 0.346, p < 0.01) and FSM2 (r = 0.233, p < 0.01) show significant positive correlations, while FSM3 has a weaker, non-significant correlation (r = 0.169).

**Table 9:** Results of the relationship of intercultural contact and intercultural competence.

	FSM	FSM1	FSM2	FSM3	FICA	FICA1	FICA2	FICA3	FICA4	FICA5	FICA6	IC
FSM	1.000	.610**	.599**	.661**	.376**	.160	.136	.189*	.311**	.175*	.175*	.387**
FSM1	.610**	1.000	.056	.121	.193*	-.017	.012	.114	.299**	.106	.090	.346**
FSM2	.599**	.056	1.000	.093	.265**	.110	.189*	.158	.126	.077	.123	.233**
FSM3	.661**	.121	.093	1.000	.246**	.204*	.056	.061	.160	.177*	.099	.169
FICA	.376**	.193*	.265**	.246**	1.000	.572**	.431**	.464**	.563**	.455**	.506**	.466**
FICA1	.160	-.017	.110	.204*	.572**	1.000	.037	.174*	.278**	.183*	.072	.271**
FICA2	.136	.012	.189*	.056	.431**	.037	1.000	.058	.074	.067	.069	.047
FICA3	.189*	.114	.158	.061	.464**	.174*	.058	1.000	-.008	-.122	.243**	.144
FICA4	.311**	.299**	.126	.160	.563**	.278**	.074	-.008	1.000	.255**	.121	.373**
FICA5	.175*	.106	.077	.177*	.455**	.183*	.067	-.122	.255**	1.000	.023	.330**
FICA6	.175*	.090	.123	.099	.506**	.072	.069	.243**	.121	.023	1.000	.299**
IC	.387**	.346**	.233**	.169	.466**	.271**	.047	.144	.373**	.330**	.299**	1.000

Note

** p< 0.01.

*p< 0.05.

Similarly, the FICA dimension strongly correlates with IC (r = 0.466, p < 0.01), showing that participation in foreign intercultural communication activities significantly boosts IC. Sub-dimensions FICA1 (r = 0.271, p < 0.01), FICA4 (r = 0.373, p < 0.01), FICA5 (r = 0.330, p < 0.01), and FICA6 (r = 0.299, p < 0.01) also show significant positive correlations. Among these, FICA4 (understanding English culture through making foreign friends abroad) has the strongest impact on IC.

Alongside the quantitative findings, qualitative insights were gathered in interviews with 16 participants to further understand the factors influencing their intercultural competence. Thematic analysis of the interview data revealed two primary themes: language proficiency and institutional support, as summarized in Table 10 below.

**Table 10:** Qualitative results for RQ2.

Themes	Subthemes	Description	Example
Language Proficiency	Language Proficiency	Language skills affecting comfort and effectiveness in intercultural interactions	“I want to interact more, but my English is so poor that I am afraid to speak”.
Institutional Support	Post-Arrival Guidance	Lack of continuous guidance abroad, leading to adaptation challenges	“The orientation before we came here was great, but once we got here, we were left to figure things out on our own”.
	Opportunities for Intercultural Engagement	Desire for structured intercultural engagement (e.g., outings, internships)	“I hope schools could organize more outings and offer internships. . .to enhance mutual understanding”.

Firstly, most participants highlighted the crucial role of language skills in managing intercultural environments. Many noted that their limited proficiency in English impeded their development of intercultural competence. For instance, one participant expressed in the interview:

I think the biggest factor is my English ability. I want to interact more with foreign classmates, teachers, and locals to improve my intercultural competence, but my English is so poor that I am afraid to speak and don’t know how to express myself. I’m worried that others will laugh at my mistakes. Many things I think about in my mind, but I don’t know how to put them into words in English.

Another participant with a low intercultural competence assessed in the questionnaire echoed this concern, adding that their language difficulties limited social opportunities:

I really want to connect with people here, but it’s hard when I can’t keep up in conversations. I feel like I’m missing out on making friends and learning from others just because of my English.

These reflections highlight how language barriers inhibited their comfort and confidence in intercultural settings, ultimately affecting their ability to build meaningful connections and improve their intercultural competence.

In addition, participants pointed to institutional support as a major factor in their intercultural experience. This theme emerged with two subthemes: post-arrival guidance and opportunities for intercultural engagement. Several students expressed a lack of ongoing support after their arrival, contrasting with the helpful pre-departure orientation. As one student noted:

The orientation we received before leaving was thorough, and it gave us a good foundation. But after we arrived, there was no one to check in or help us adjust. I faced so many cultural shocks and was often confused, but I had to figure everything out by myself.

Others shared similar sentiments, wishing they had guidance during crucial adjustment periods. One participant recounted their experience of feeling isolated and needing someone to explain social customs:

I had no idea about local customs, and I was constantly worried I was being rude without knowing it. If someone from the school could guide us or answer questions along the way, it would have made a huge difference.

In addition to guidance, many participants also expressed a strong desire for structured intercultural engagement opportunities, such as organized outings or field-related internships. They felt that these experiences could provide more authentic contexts for cultural exchange, enhancing their intercultural skills in practical settings. One participant suggested:

I wish the school here would organize more activities to help us meet people from different backgrounds. Even a simple outing or group activity would allow us to interact naturally. It’s not easy to make friends on our own, especially when we don’t know where to start.

Another participant emphasized the value of field-specific internships:

Internships related to our studies would help us connect with locals and understand their work culture. We can only learn so much in the classroom, but these experiences could give us the skills to handle real-world situations.

To conclude, the qualitative findings underscore the impact of language proficiency and institutional support on students’ intercultural experiences. The challenges they face in language and their need for more guided intercultural exposure illustrate areas where further support could enhance their competence and confidence in intercultural settings.

## 5 Discussion

A divergence between the quantitative and qualitative findings emerged regarding Chinese participants’ intercultural competence. Quantitative data suggested high levels of competence, while qualitative data indicated a more moderate level. This discrepancy may be due to differences in data collection methods. Participants may provide more positive self-assessments in questionnaires, while interviews, being more personal and interactive, could lead to more cautious responses [66]. Cultural factors, such as the Chinese emphasis on humility, might also result in under-reporting of competencies during interviews [67,68].

Despite these differences, both data sources indicate a moderate level of knowledge about Irish culture and intercultural skills, attributed to limited exposure to diverse cultural interactions. Participants mostly interacted with fellow Chinese students rather than engaging with locals. This finding aligns with previous studies [69,70] showing that Chinese students abroad often form social clusters with their compatriots, limiting intercultural engagement.

This study also examined the impact of gender, intercultural training, language proficiency, and intercultural contact on intercultural competence. Contrary to studies reporting gender-based differences [19,33], this study found no significant gender differences. This may be due to the standardized exposure to foreign cultures and teaching methods in Sino-Foreign Cooperative Education Programs, which provides equal opportunities for both genders to develop intercultural competence [71].

The study highlights the positive impact of intercultural training on intercultural competence, confirming many prior research [72,73]. However, participants reported a lack of sustained institutional support once abroad, limiting the effectiveness of the initial training. To improve this, institutions could establish stronger support networks, such as intercultural advisors or mentors [74], both at home and abroad. These advisors could offer ongoing guidance, help students cope with cultural adjustments, and facilitate connections with local communities. Additionally, intercultural training could include post-arrival workshops or online modules [75] to reinforce initial training and address real-time challenges.

The study confirms a strong positive correlation between language proficiency and intercultural competence, consistent with existing research [34–36]. Higher language skills were key to effective communication, boosting confidence and enabling meaningful engagement in diverse cultural settings. This supports the notion that advanced language proficiency is essential for understanding cultural nuances and actively participating in intercultural exchanges [76]. The study also suggests, in line with prior research [77,78], that intercultural programs should prioritize ongoing language training to further enhance participants’ ability to engage effectively in intercultural interactions.

Lastly, the study identifies a significant positive relationship between intercultural contact experiences and levels of intercultural competence. Activities such as making foreign friends and participating in internships or part-time jobs abroad were particularly influential. Many participants expressed a desire for greater institutional support in securing internship opportunities, highlighting a gap in the current support structures, as noted in previous studies. Addressing this need could significantly enhance students’ intercultural competence by providing valuable real-world experiences that complement academic learning.

## 6 Implications

This study offers valuable insights for institutions, policymakers, and students involved in Sino-Foreign Cooperative Education Programs. For institutions, the findings highlight the need for continuous support systems to maximize the benefits of dual-institutional frameworks. Support should go beyond initial training to include ongoing guidance, resources, and interventions to help students overcome cultural challenges and adapt to new environments. Enhanced language training, covering both conversational skills and cultural nuances, should also be integrated into the curriculum to promote meaningful interactions and build confidence.

For policymakers, the study underscores the importance of improving program design and implementation. Strengthening institutional collaborations, ensuring sustained support during students’ time abroad, and fostering partnerships with local employers to create more internships and job opportunities are key steps toward building a sustainable international education framework.

For students, the study highlights the benefits of actively engaging in intercultural opportunities, such as forming local friendships and participating in internships or part-time jobs. These experiences are crucial for developing intercultural competence and enriching their educational journey. Students should also utilize institutional resources to address cultural challenges and support their adaptation process.

However, this study is limited by its focus on 130 Chinese students in Ireland, which may affect the generalizability of the findings. Students in other countries under similar programs may face different experiences that influence their intercultural competence. Future research should include a broader, more diverse sample across various host countries to provide a more comprehensive understanding of intercultural competence in different contexts.

## Supporting information

S1 File Questionnaire. (PDF)

S2 File Minimal data set. (XLSX)
