# Supplementary material for: Intercultural competence of Chinese students abroad: An investigation under Sino-foreign Cooperative Education Programs
Source: PLoS One. 2025 Feb 5;20(2):e0316937. doi: 10.1371/journal.pone.0316937 (PMC11798469; doi:10.1371/journal.pone.0316937)
Supplement: S1 File — (PDF) [file pone.0316937.s001.pdf]

Questionnaire

Part I Background Information

Thank you for your time and participation in the project! The first spart of the questionnaire is designed to get background information of participants. It is noted that all the information you fill in will be used only for research and publication purposes and used anonymously.

1. Name: ________________ Age: ___________

2. Gender: (A) Male (B) Female

3. Grade: (A) First-year (B) Second-year (C) Third-year (D) Fourth-year

4. English test score: IELTS______ TOEFL _______ Duolingo______ Other______

5. Do you have any intercultural training experience? If yes, what is it?

Part II Assessment of Intercultural Competence of Chinese College Students

This section is designed to collect your self-evaluation of your intercultural competence. According to your understanding, rate the following items from 1 to 5 about your objective and universal intercultural competence. We need your true thoughts. Thank you!

Please read the following items and rate yourself using the scale below:

(A) very low (B) low (C) average (D) high (E) very high

1. Knowledge of self

(1) understanding native history

(2) understanding native social norms

(3) understanding the native sense of values

2. Knowledge of others

(1) understanding foreign knowledge of history

(2) understanding foreign social norms

(3) understanding the foreign sense of values

(4) understanding foreign cultural taboos

(5) understanding foreigners' speech

(6) understanding basic concepts of intercultural communication

(7) understanding successful intercultural communication strategies

3. Attitudes

(1) willingness to learn from those who differ from one's self and culture

(2) willingness to respect foreigners' lifestyles and customs

(3) willingness to learn foreign languages and cultures well

4. Intercultural communicative skills

(1) the skill of consulting with foreigners when misunderstandings occur

(2) the skill of communicating with foreigners using body language or other nonverbal communication when it is difficult to communicate using language

(3) the skill of successfully communicating with foreigners

(4) the skill of treating foreigners politely

(5) the skill of avoiding offending foreigners with inappropriate words and behavior

(6) the skill of avoiding prejudice against foreigners

(7) the skill of avoiding violating foreigners' privacy

(8) the skill of having intercultural sensitivity

(9) the skill of understanding different perspectives when encountering different cultural affairs

5. Intercultural cognitive skills

(1) the skill of acquiring knowledge of other cultures from foreigners

(2) the skill of learning intercultural communication strategies

(3) the skill of learning how to manage cultural conflicts

6. Awareness

(1) realizing cultural differences and similarities when communicating with foreigners

(2) realizing the differences in cultural identity when communicating with foreigners

(3) judging cultural situations from both one's own and the other's cultural perspective

Part III Intercultural Contact Scale

This section is designed to collect data on your intercultural contact experiences. Please choose the most appropriate ones based on the frequencies of your engaging in the following intercultural contact activities.

1. Foreign Social Media (FSM)

(1) text communication with English native speakers through social media abroad

Never *Rarely* *Sometimes* *Often* *Always*

(2) voice communication with English native speakers through social media abroad

Never *Rarely* *Sometimes* *Often* *Always*

(3) video communication with English native speakers through social media abroad

Never *Rarely* *Sometimes* *Often* *Always*

2. Foreign Intercultural Communication Activity (FICA)

(1) understanding English culture through completing an internship or part-time job abroad

Never *Rarely* *Sometimes* *Often* *Always*

(2) understanding English culture through participating in an international academic conference abroad

Never *Rarely* *Sometimes* *Often* *Always*

(3) understanding English culture through attending courses abroad

Never *Rarely* *Sometimes* *Often* *Always*

(4) understanding English culture through making foreign friends abroad

Never *Rarely* *Sometimes* *Often* *Always*

(5) understanding English culture through lving in a homestay familiy abroad

Never *Rarely* *Sometimes* *Often* *Always*

(6) understanding English culture through participating in volunteer activities abroad

Never *Rarely* *Sometimes* *Often* *Always*
